# Disentanglement of Cape fur seals (*Arctocephalus pusillus pusillus*) with reversible medetomidine-midazolam-butorphanol

**DOI:** 10.4102/jsava.v92i0.2119

**Published:** 2021-05-21

**Authors:** Brett R. Gardner, Brandon Spolander, S. Mduduzi Seakamela, Steven A. McCue, Pieter G.H. Kotze, Maryke Musson

**Affiliations:** 1 Werribee Open Range Veterinary Hospital, Zoos Victoria, Werribee, Australia; 2 Aquavet Africa, Hermanus, South Africa; 3 Department of Forestry and Fisheries, Department of Forestry, Fisheries and Environment, Cape Town, South Africa; 4 Department of Education Foundation, Two Oceans Aquarium, Cape Town, South Africa

**Keywords:** reversible anaesthesia, immobilisation, Cape fur seal, medetomidine, midazolam, butorphanol, disentanglement, atipamezole, naltrexone, *Arctocephalus pusillus pusillus*

## Abstract

Anaesthesia in pinnipeds is considered a much higher risk than in most terrestrial mammals because of their frequent proximity to water and physiological and anatomical adaptations related to diving, which also influence their anaesthesia management. Anaesthetising and immobilising entangled seals does not allow for selection of animals that are at a safe distance from the water’s edge. Medetomidine-midazolam-butorphanol (MMB) sedation was trialled on eight entangled Cape fur seals (CFS) (*Arctocephalus pusillus pusillus*) to determine if it was safe to use on animals that entered the water post-darting. The MMB was given at an estimated dose of 0.03 mg/kg, 0.2 mg/kg and 0.2 mg/kg, respectively, via remote darting. Sedation was reversed with intramuscular atipamezole (0.15 mg/kg) and naltrexone (0.4 mg/kg) to antagonise the effects of medetomidine and butorphanol, respectively. Moderate sedation was achieved in six animals. Six of the animals entered the water after being darted. There was a single mortality and a single animal that was too lightly sedated for capture. The preliminary results indicate that MMB produces suitable sedation for disentanglement of CFS. Additionally, MMB might be suitable for application to field-based biological research.

## Introduction

Sedation and anaesthesia of otariid seals has advanced significantly in the last decade, producing safer outcomes for these marine mammals (Haulena & Schmitt 2018). Historically, pharmacological immobilisation of otariid seals has been associated with a high rate of mortality (Geschke & Chilvers 2009). Much of this was attributed to the presence of water near sedated animals and their specific physiological adaptations to diving (Baylis et al. 2015; Haulena & Schmitt 2018). Despite these advances there are still significant challenges associated with immobilisation of wild otariids.

Along the South African coastline and especially within its harbours, marine wildlife is exposed to large amounts of anthropogenic waste. Cape fur seals (CFS) (*Arctocephalus pusillus pusillus*) interact with human activities at harbours along their range, most notably on the west coast (south-east Atlantic Ocean). These interactions are most evident at fishing harbours such as Cape Town and Hout Bay. Fishing harbours present haul-out and scavenging opportunities for CFSs, including interactions with anthropogenic debris. Because of their inquisitive and explorative nature, CFSs are at risk of cervical entanglements in marine debris. Worldwide, anthropogenic marine debris poses a health risk to marine mammals. In pinnipeds, this debris often originates either directly or indirectly from the fishing industry (Lawson et al. 2015; Page et al. 2004). Unpublished disentanglement data (1990–2008) from the Victoria & Alfred (V&A) Waterfront (Cape Town) revealed that the majority of seal entanglements were from fishery products such as raffia cord, polypropylene box bands and monofilament fishing line (Department of Environment, Forestry and Fisheries [DEFF], unpublished data).

The DEFF initiated a CFS disentanglement programme in 1998, which is now run collaboratively with Two Oceans Aquarium at the V&A Waterfront in the Cape Town Harbour. Ad hoc interventions have been conducted since the 1980s. Over 3000 CFSs have been disentangled since 1984, more than 1000 of these between 2008 and 2019. Historically, disentanglement efforts were conducted on restrained conscious and unaware resting CFSs. Hoop nets were used to catch and restrain animals within the V&A Waterfront. Curved blades with a blunt outer curve and an inner cutting blade, attached to a long pole, were used to cut entanglements where capture and restraint were not feasible. Limitations to the operations include CFSs that are out of the reach of these two removal techniques, are too large to handle safely or are too alert, taking flight before successful disentanglement is possible. This comprises approximately 10% of entanglement cases. Additionally, responders have been bitten during these operations. There is significant risk of traumatic damage from a CFS bite and additionally a potential zoonotic risk of infection for these staff members. Pinnipeds are known to carry various bacteria in their oral cavity, such as *Mycoplasma* sp., that cause seal finger and other infectious conditions (Hunt et al. 2008). It is also a reasonable assumption that the procedure of capture and manual restraint produces a significant stressful incident for the CFSs.

It is becoming increasingly important not just within South Africa but worldwide to have a safe and reliable means of remotely sedating otariid seals for rescue, rehabilitation, animal welfare and research purposes. Sedation has been proven as a safe, reliable means for disentanglement of seals (Frankfurter, DeRango & Johnson 2016; Haulena & Schmitt 2018). There is minimal literature available on remote drug delivery to CFSs. Much of the literature available on their immobilisation is dated and associated with poor predictability, older anaesthetic drug combinations and high mortality rates (David et al. 1988). After being trapped, South American fur seals (*Arctocephalus australis*) have been successfully sedated with a combination of midazolam and either ketamine or medetomidine (Katz, Reisfeld & Franco-Trecu 2019). New Zealand sea lions (*Phocarctos hookeri*) have been successfully darted with tiletamine-zolazepam (TZ), but the technique was used only on large sleeping or resting animals more than 50 m from the water’s edge (Geschke & Chilvers 2009). New Zealand fur seals (*Arctocephalus forsteri*) have been successfully darted with TZ, with some animals entering the water and a larger than expected proportion of animals surviving entry into the water (McKenzie et al. 2013).

Despite the lack of veterinary guidelines on how best to proceed with remote drug delivery in otariids, it is important to investigate different techniques or improve on existing techniques. Drowning is one of the most significant risk factors during remote immobilisation of seals, particularly when wild animals of unknown health status are darted in an uncontrolled environment (Baylis et al. 2015). This is especially true for disentanglement efforts at the V&A Waterfront in the Cape Town Harbour, where the darted animal will almost invariably enter the water.

Successful immobilisation with improved safety margins has been reported in California sea lions (CSLs) (*Zalophus californianus*), using medetomidine-midazolam-butorphanol (MMB) in both captive and wild sea lions, producing reliable sedation without compromising respiration (Frankfurter et al. 2016; Melin et al. 2013; Spelman 2004). This drug combination has been used in CSLs under conditions where animals entered the water post-darting and still maintained effective respiration. Most of the animals in that study that entered the water maintained spontaneous breathing and were safely retrieved post-darting or sedation (Frankfurter et al. 2016). To the authors’ knowledge, this immobilisation drug combination has never been used on wild CFSs; this is the first report of its use under these circumstances at the V&A Waterfront Cape Town Harbour and the Hout Bay Harbour.

## Materials and methods

### Animals and area

Entangled CFS were identified by the disentanglement team during routine checks within the V&A Waterfront or by members of the public. Darting was conducted at two fishing harbours situated in the Cape Peninsula, South Africa: V&A Waterfront in Cape Town and Hout Bay. A total of eight CFSs were darted for this trial: V&A Waterfront (*n* = 6) and Hout Bay (*n* = 2). Estimated weights ranged from 35 kg to 120 kg with a median weight of 82.5 kg. Six seals were males, with one female and one of unknown sex.

### Dart projector and remote drug delivery darts

Cape fur seals were darted using a Pneu-Dart X-Caliber CO_2_ dart rifle with either a 1-mL or 2-mL type-P dart with a 2.54-cm, barbed triport needle (Pneu-Dart, Williamsport, PA, United States). Barbed darts facilitated visual identification of the sedated individual once in the water and amongst other CFSs. The gluteal muscles were used as a target area because of the lack of substantial subcutaneous adipose covering of muscle in this region, the ease of darting into this location and the ability to see the dart as the animal dives under water.

### Immobilisation and reversal agents

Once an animal was identified, four staff members experienced in working with CFSs individually estimated the weight of the animal. An average between these four estimates was calculated. Animals were darted according to this weight with 0.03 mg/kg medetomidine (20 mg/mL; Kyron Laboratories, Benrose, South Africa), 0.2 mg/kg midazolam (50 mg/mL, Dazonil; Wildlife Pharmaceuticals, White River, South Africa) and 0.2 mg/kg butorphanol (50 mg/mL; Kyron Laboratories, Benrose, South Africa). The medetomidine and butorphanol were antagonised intramuscularly using atipamezole 0.15 mg/kg (5 mg/kg, Antisedan; Zoetis, Sandown, South Africa) and naltrexone 0.4 mg/kg (50 mg/mL, Trexonil; Wildlife Pharmaceuticals, White River, South Africa), respectively, both administered intramuscularly. In one animal there was a shortage of naltrexone, and naloxone was substituted (0.4 mg/mL, Narcan; Fresenius-Kabi, Midrand, South Africa) at 0.03 mg/kg, then repeated once at 0.015 mg/kg 7 min later, and a final dose of 0.005 mg/kg 15 min after the first reversal, all administered intramuscularly. As none of the animals were weighed, all dosages were based on estimated weights. Vital parameters including respiratory rate, heart rate, capillary refill time and depth of sedation were monitored where possible.

### Boats and divers

Two rigid inflatable boats with a solid hull and inflatable outer pontoon were used. The smaller boat, a Gemini Waverider 470, is fitted with a F15 Yamaha outboard motor and the larger boat, a Gemini Waverider 850, with two Yamaha F200 engines. The smaller boat, which is more manoeuvrable and able to get into more difficult spaces within the close confines of the marina and harbour, serves as the platform from which the darts are delivered if this is not possible from shore. The larger boat, which sits higher above the water and is faster, is used as a mobile observation post and workstation for retrieving sedated animals from the water. It has sufficient deck space to process entangled seals. Seals are processed on board the boat when it is not possible to process them on shore. When seals are darted from shore, both boats are placed on standby in the water, irrespective of whether the animal enters the water. Each boat has a commercial diver on board and is equipped with a variety of aquatic retrieval equipment for hauling and restraining sedated seals from the water.

### Ethical considerations

This work was done in accordance with a permit issued by the South African Department of Forestry, Fisheries and Environment, permit number RES2019/81, amendment 2. Ethics clearance for the procedure was approved by the Ethics Committee of the Two Oceans Aquarium.

## Results

Seven of the CFSs in this study were located within 1.5 m or less from the water’s edge. The remaining CFS was less than 5 m from the water’s edge. Six of eight darted CFSs entered the water, two immediately and the other four 3 min – 4 min post-darting. The two individuals that remained out of the water after being darted were large males.

Three of the six CFSs that entered the water remained within approximately 25 m from where they had been darted. The other three moved away to distances of approximately 60 m, 135 m and 175 m from where they were darted. On average CFSs took 4 min 3 s to show initial signs of effect post-darting. The average time from darting to handling of the entangled CFS was 20 min 29 s. The average recovery time after administration of the immobilisation reversal agent was 26 min 35 s.

**FIGURE 1:**
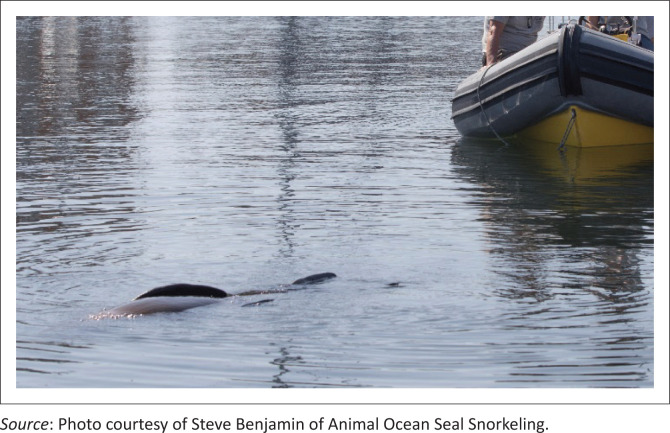
A sedated fur seal ready to be retrieved by boat.

Animals were deemed suitably sedated for retrieval from the water with a modified hoop net when they stopped swimming and became stationary on the surface, either on their back or floating laterally (Figure 1). They were breathing regularly and would lift their head out from under the water to take a breath before resubmerging it. Occasionally the stimulation of being retrieved from the water with the net caused CFSs floating in sternal recumbency to attempt diving. These dive attempts were mostly subsurface with animals being visualised as they left a bubble stream from their nostrils and then resurfaced a few meters from the dive entry, allowing them to be retrieved by net.

Two CFSs received a supplementary dose of immobilisation drugs. One was showing initial drug effects but was continually stimulated by adjacent non-drugged individuals and deemed unsafe to approach. It was given 50% of the initial induction dose of all three drugs. The other seal was initially darted but the dart bounced off the animal on impact. The degree of drug delivery in this individual was unknown and it was therefore observed for an additional 15 min. After showing no signs of effect by 15 min from initial darting, a second dart was delivered with a full induction dose. The animal disappeared from the surface 15 min after the second dart and was not located despite two divers entering the water to retrieve the animal. The body was found floating close to where it had originally disappeared 5 days after the incident. An independent necropsy was inconclusive as to the cause of death.

Six of the eight seals were successfully retrieved with relative ease once 20 min had elapsed from the time of darting. One seal was too lightly sedated for capture and by 53 min post-darting was deemed fully recovered without the administration of reversal agents.

Recovery after administration of reversal agents was predictable and reliable in all instances other than the single case in which naltrexone was substituted with naloxone.

## Discussion

Comparing the published literature for the MMB combination in otariid seals, it appears that the midazolam and butorphanol dose is fairly consistent throughout its use in captive CSLs (Spelman 2004), wild CSLs caught in haul-out traps and then manually injected (Melin et al. 2013) and wild CSLs darted with MMB (Frankfurter et al. 2016). Midazolam doses ranged between 0.15 mg/kg and 0.25 mg/kg. The lowest dose rate was recorded for wild CSLs caught in haul-out traps (Spelman 2004). Similarly, the butorphanol dose was fairly consistent across all three of these studies. The recorded dose rate ranged from 0.1 mg/kg in CSLs caught in haul-out traps (Melin et al. 2013) to 0.2 mg/kg for CSLs darted remotely (Frankfurter et al. 2016). In captive CSLs a range of 0.2 mg/kg – 0.4 mg/kg was reported (Spelman 2004). The major difference is in the medetomidine dose when comparing captive to wild CSLs, irrespective of manual injection in haul-out traps or darting remotely, when compared to captive animals. Captive CSLs were administered 0.01 mg/kg – 0.013 mg/kg medetomidine (Spelman 2004) compared to 0.03 mg/kg in wild CSLs (Frankfurter et al. 2016; Melin et al. 2013). Whether this difference relates to the stress response between captive versus wild CSLs and the effect of adrenaline on the α2 adrenoreceptor, thereby affecting medetomidine, would require further detailed studies. It is well documented that wild animals require higher doses compared to captive animals (Eggers et al. 2020). Our dose rates were based on those reported for wild remotely darted CSLs (Table 1).

**TABLE 1:** Doses of anaesthetic agents given to the Cape fur seals during the investigation (*N* = 8).

Event no.	Animal identification no.	Gender	Estimated weight (kg)	Medetomidine dose (mg)	Midazolam dose (mg)	Butorphanol dose (mg)	Atipamezole dose (mg)	Naltrexone dose (mg)
1	YY0080/0081	M	70	2.2	16.0	21	35	7.8
2	YY0082/0082	F	35	1.1	5.3	7	14	5.3
3	003	M	40	1.2	8.0	12	-	-
4	Y0084/U7947	M	120	4.2	24.0	36	72	21.0
5	005	M	90	2.7	18.0	27	-	-
6	006	U	95	3.0	20.0	30	-	-
7	007	U	85	2.6	17.0	17	8	12.8
8	008	M	80	2.4	16.0	16	32	12.0

M, male; F, female; U, unknown.

A mortality rate of 12.5% was recorded in our study, with a sample size of only eight animals, making comparisons with other studies more difficult. In CSLs using the same dose range with MMB, under conditions where the animals were able to access the water post-darting, there was a 20% mortality rate; this study also had a small sample size of 15 animals (Frankfurter et al. 2016). A known mortality rate of 18.2% was reported in the scant literature reporting remote darting of CFSs; various combinations of ketamine, carfentanyl, xylazine, azaperone and droperidol were used (David et al. 1988). In that study success at the retrieval of sedated and partially sedated animals was only 54.6%. Animals far away from the water (> 30 m – 40 m) were selected to avoid the chance of them re-entering the water post-darting. Despite these measures, the retrieval rate was poor compared to the 100% retrieval rate after remote darting of CSLs using MMB (Frankfurter et al. 2016). The latter study included the use of telemetry, which appeared to greatly improve the retrieval rate. Our study had a retrieval rate of 75% on the day. The use of MMB appears to be safer as it does not produce deep sedation at the dosages used, therefore reducing the risk of drowning. Entangled animals routinely appeared to be more skittish and often remained near the water’s edge, resulting in animals more likely to enter the water post-darting, thus necessitating a drug combination with a significantly decreased drowning risk. Irrespective of the immobilising drug combination used, the fact that animals need to be darted produces variability in the successful delivery of the immobilising drugs. Because of MMB producing mild to moderate sedation at the doses used, there is a risk of not achieving suitable sedation compared to other drug combinations that produce deep sedation or anaesthesia. One of the animals was recovered dead 5 days post-darting but is not included in the retrieval rate. Had telemetry been employed, this animal would probably have been recovered by a diver soon after disappearing from the surface. It would be impossible to determine if the outcome of survival would have been any different as the necropsy of this animal was inconclusive. In the case of the other animal that was allowed to recover spontaneously, telemetry could perhaps have increased the efficiency with which this animal was tracked. Thus, the time since the initial effects would have been decreased, allowing this animal to be retrieved whilst still suitably sedated.

Seventy-five percent of the animals in our study entered the water after being darted. Had a less appropriate immobilisation combination been used, the mortality rate could be expected to be higher. A study of remote darting of CSLs for the purpose of disentanglement reported return to water rates of 87% (Frankfurter et al. 2016). Data from 32 studies on Southern hemisphere otariids indicated that 89.9% of the mortalities occurred as a result of complications during anaesthesia: 22.2% during the initial induction and capture of the animals and 66.1% during the maintenance of anaesthesia (Baylis et al. 2015). In an extensive study on New Zealand fur seals, only 13.3% of animals darted with TZ escaped back to the sea. Most of these animals were more than 20 m from the water’s edge at the time of darting. Of the 13.3% that returned to sea, 37.5% had an unknown outcome once at sea, potentially as mortalities. The other 62.5% breathed satisfactorily at the surface for an extended period prior to recovery (McKenzie et al. 2013). The effects of TZ on respiration and mentation are more profound than those of MMB (Haulena & Schmitt 2018). Our study in CFSs and a recent study in CSLs using MMB (Frankfurter et al. 2016) reported mortalities only during the initial stages of induction, with stable sedation once the animals were captured and restrained. Potentially, the mortality risk may be decreased using MMB as it does not routinely produce deep sedation. This allows the animal to breathe spontaneously when lifting its head from the water, whilst maintaining a protective reflex of the larynx when the head is submerged.

As most of the CFSs in our study remained mildly to moderately reactive and the main objective was disentanglement, physiological anaesthetic data such as heart and respiratory rates were not collected in all but two animals. Future studies on larger numbers of animals would be worth pursuing to determine effective dosages according to exact weights with detailed physiological parameter monitoring.

The use of MMB reversible anaesthesia in otariid seals has applications beyond mere disentanglement. Most likely its use would be ideal to situations where there is a high likelihood of animals entering the water post-darting, whether this be in field research, relocating problem animals or in areas with difficult access for manual capture.
